# Two Sisters From Qatar With TUSC3 Genetic Mutation: Psychiatric Considerations

**DOI:** 10.7759/cureus.17616

**Published:** 2021-08-31

**Authors:** Yahia Albobali, Manar Y Shahwan, Mahmoud Y Madi, Saleem Al-Nuaimi

**Affiliations:** 1 Psychiatry, Hamad Medical Corporation, Doha, QAT; 2 Internal Medicine, University of Jordan, Amman, JOR; 3 Internal Medicine, University of Kansas Medical Center (KUMC), Kansas City, USA

**Keywords:** autosomal recessive disorders, clinical psychiatry, intellectual disability, psychoeducation, psycho-diagnostics & therapy

## Abstract

Defects in the tumor suppressor candidate 3 (TUSC3) gene have been identified in individuals with autosomal recessive intellectual disability (ARID). Our report on two sisters from Qatar with a mutation in the TUSC3 gene focuses on the behavioral manifestations and management provided to them. The sisters, daughters of consanguineous parents, exhibited aggressive and impulsive behavior, along with hyperactivity and emotional dysregulation. They also exhibited abnormal sleep and eating patterns. Behavioral therapy and psychotropic medications including aripiprazole 3.75mg, clonidine 0.025mg, and guanfacine 1mg were used for the management of aggressive and agitated behavior. The two girls showed a reduction in aggressive behavior, hyperactivity, impulsivity, and insomnia in response to 2mg daily of guanfacine. Few families around the world were reported to have mutations in the TUSC3 gene resulting in intellectual disability. We describe the first two reported cases of TUSC3 gene mutation in Qatar. We encourage further research to study the effects of TUSC3 gene mutation, its manifestations, and treatment.

## Introduction

Tumor suppressor candidate 3 (TUSC3) was first discovered in humans in 1996. It is located on chromosome 8p22. It was found to be expressed in different areas in the body, including prostate, lung, colon, liver, ovary, testis, placenta, and adipose tissue [1]. TUSC3 constitutes a major component in cellular magnesium transport [2], which plays an important role in learning and memory [3]. A deletion or mutation in the TUSC3 gene is associated with intellectual disability [4-10]. TUSC3 may act as a tumor suppressor gene and its mutations have been implicated in malignant transformation [11].

As research data accumulates, hope for therapeutic targeting of TUSC3 protein to treat psychiatric and oncological diseases may be on the horizon. Therapeutic strategies may include recombinant gene therapy, targeting negative regulators of TUSC3, and targeting destabilized pro-oncogenic TUSC3 mutants. There are more than 500 genes that have been linked to intellectual disability [12]. In this report we describe the psychiatric and behavioral management of two Qatari sisters who have TUSC3 gene mutation and intellectual disability. Our goal is to shed light on this important genetic mutation and its psychiatric implications.

## Case presentation

The two sisters accessed the child and adolescent mental health services for the first time in September 2018. They were nine and twelve years old at the time. They had a known prior history with the child development center due to developmental concerns but no known prior psychiatric or mental health assessment. They were referred to our service due to severe disruptive behaviors. They underwent a psychiatric assessment initially, followed by psychological and occupational therapy assessments. For both the girls, the main concerns reported by their mother included severe aggressive behaviors, severe impulsivity (running away from home, running into traffic, turning the gas stove on in the kitchen and leaving it unattended), hyperactivity, obsessive behaviors (getting stuck on things, difficulties with transitions), and emotional dysregulation (mood swings, temper tantrums, intense periods of anger).

The two girls are the result of consanguineous marriage - the father and mother are first cousins. There were no reported complications during pregnancy, labor, or delivery of the girls. However, their mother noted that both had excessive crying during their first year of life. There were significant delays in developmental milestones in both motor and speech areas. Both girls suffer from motor and language delays, with the majority of their speech being unintelligible. They lack the ability to read and write. The sisters attend a school for children with special needs. They have no ophthalmologic or otolaryngological abnormalities. Other physical and clinical features are presented below (Table 1).

 

**Table 1:** Other clinical features

	Older Sister	Younger Sister
Age	13 years	10 years
Height	148.2 cm	130 cm
Weight	45.9 kg	25.05 kg
IQ	40 (2010)	Not available
Dysmorphic features	Deep-set eyes	Deep-set eyes

The sisters have other siblings, including a brother and a sister who do not have any known developmental or psychiatric abnormalities. However, the mother does describe academic difficulties with her son. This was not brought to medical attention and he is in a regular classroom setting. The family history is significant for suspected intellectual disability and behavioral disturbances in two cousins from the maternal side. They include one male and the other female. They are the children of the mother’s brother. They have not had any medical workup or confirmed diagnoses. The mother describes the two cousins as being similar in presentation to her two daughters described in this case report.

Investigations

Chromatography of plasma amino acids, urine organic acids, lactic acid, and creatine kinase were done and showed no abnormalities. Genetic testing for the two sisters was done on December 13, 2013. Using genomic DNA from the submitted specimens, the SureSelect XT2 All Exon V4 kit (Agilent Technologies, Santa Clara, CA, USA) was used to target the exon regions of the genomes. These targeted regions were sequenced using the HiSeq 2000 sequencing system (Illumina, San Diego, CA, USA) with 100bp paired-end reads. The DNA sequence was mapped to and analyzed in comparison with the University of California, Santa Cruz (UCSC) published human genome hg19 reference sequence. The targeted coding exons and splice junctions of the known protein-coding Reference Sequence (RefSeq) genes were assessed for the average depth of coverage and data quality threshold values. The XomeAnalyzer (GeneDx, Gaithersburg, MD, USA) was used to evaluate sequence changes in these two individuals compared to other sequenced family members. All reported sequence variants in the proband and relative samples were confirmed by conventional di-deoxy DNA sequence analysis or other appropriate methods. The test showed that the two girls have a homozygous TUSC3 gene mutation, S343T variant, coding DNA: c.1028 G>C. Genetic testing was done for the parents and showed that they are both heterozygous for the above mutation. The brother was tested as well and he did not have the mutation.

Management

Initial management of these two sisters consisted of parent psychoeducation about their condition, expected course, and treatment options. We reinforced the importance of behavioral management, as the two girls’ behaviors were clearly distressing to the parents. The parents were educated about what to expect from the medications, their effects, side effects, and the risk/benefit of therapeutic interventions. Initially, the mother did not want any psychotropic medications. Therefore, both sisters were referred to psychology and occupational therapy for further assessment and management.

However, due to ongoing behavioral and emotional dysregulation, the mother consented to the use of medications. The older sister was started on aripiprazole to manage her irritability and violent behavior. She was started on 3.75mg. The reason for starting at this dose is due to the formulation availability at our institute. Unfortunately, she developed significant sedation as well as abdominal discomfort. Despite the side effects, aripiprazole substantially reduced her aggression, impulsivity, and hyperactivity. However, the mother discontinued the medication and did not want to trial any other anti-psychotics, anti-depressants, or mood stabilizers due to concerns of side effects. The mother also had concerns regarding the stigma associated with putting her daughters on psychotropic medications.

Therefore, we discussed the use of alpha-2-agonists as a potential trial. Their mother consented to a trial of clonidine after its mechanism of action was elucidated. The older sister was started on 0.025mg of clonidine daily. The dose was increased gradually to 0.05mg twice daily. It was not effective in reducing aggressive behavior but helped improve her sleep. Therefore, we switched her from clonidine to guanfacine extended-release. She was started on 1mg daily and was increased to 2mg at night. Guanfacine reduced aggressive behavior significantly and improved sleep without causing daytime drowsiness. She has now been on guanfacine extended-release for one year and remains stable with sustained benefits and minimal sedation.

The younger sister was less violent and hence was not started on aripiprazole like her older sister. Instead, she started on clonidine 0.025mg daily. The dose was increased gradually to 0.05mg twice daily. It was not effective in reducing aggressive behavior and did not improve her insomnia. She was then switched to guanfacine extended-release. She was started on 1mg daily and increased to 2mg daily at night. Guanfacine significantly reduced her hyperactivity and impulsivity but caused slight daytime sedation. She has now been on guanfacine extended-release for one year and remains stable with sustained benefits and improved sedation side effects. The mother brought up concerns regarding ongoing inattention, hyperactivity, and distractibility. Unfortunately, the guanfacine extended-release did not significantly improve these symptoms. The mother agreed to add on other medication. We discussed the use of stimulants vs. non-stimulants. We agreed to a trial of atomoxetine after a discussion of risks and benefits. And so the younger sister was started on a low dose of 10mg daily and slowly titrated up to 35mg daily. Her mother reports a noticeable improvement in the patient’s ability to sustain attention, be less distractible, and be more engaged with tasks. The sisters will continue to have frequent follow-ups to monitor the response to medications and therapy, and to check for any side effects of the medication.

## Discussion

The prevalence of consanguineous marriages in Qatar is high. A cross-sectional study done in 2006 in Qatar estimated the prevalence of consanguinity in 54% of the population [13]. This report and similar reports of genetic mutations resulting from consanguineous marriages in the Gulf countries and other Arab countries in general clearly reveal the need for genetic health education and implementation of culturally sensitive technologies, including prenatal screening and pre-implantation genetic counseling. This may offer hope to families burdened with genetic mutations in this area of the world where consanguinity is rooted in cultural traditions.

Few families around the world have been identified with TUSC3 mutation causing intellectual disability. This is the second reported family from the Middle East after an Omani family. The affected sisters described in our report did not develop tumors, but it is necessary to monitor them for increased tumor risk in the future as the gene is implicated in the pathogenesis of certain malignancies.

Previous reports have shown that individuals affected with TUSC3 mutation suffer from varying degrees of intellectual disability in addition to certain dysmorphic features, which is consistent with our report [14].

Both sisters showed improvement in their behaviors when guanfacine was used. Guanfacine is an alpha-2 receptor agonist that is effective and safe in the treatment of children with attention-deficit/hyperactivity disorder (ADHD) [15]. It has also shown promise in improving disruptive and impulsive behaviors [16]. Guanfacine is the most selective alpha-2A agonist, which may explain the superiority of guanfacine over clonidine in the case of the two sisters, as clonidine is poorly selective for the alpha-2A receptors [17]. It also explains the enhanced side effect profile, as selectivity causes less sedation and blood pressure lowering effects than clonidine. The younger sister responded reasonably well to atomoxetine, which is a non-stimulant used in the treatment of children with ADHD as well.

Unfortunately, the mother did not report any significant improvements with the behavioral modifications and psychotherapeutic support provided by psychology and occupational therapy. This can be attributed to the lack of intensive programs offered by our service, as we are restricted to outpatient services only. There is also no outreach or in-home service capacity. This was a major challenge since the mother struggled with implementing any of the behavioral strategies in the home setting.

It is important to think about the long-term management plan in the case of our patients. From the psychological aspect, there has been a focus on psychoeducation for the parents and involving a specialized therapist to provide behavioral therapy to the two sisters.

## Conclusions

In this report, we described behavioral manifestation of TUSC3 gene mutation and associated intellectual disability in two sisters. Extended-release guanfacine provided an observable improvement in managing both the girls' aggressive behavior, hyperactivity, impulsivity, and insomnia. Continuous psychosocial support might help improve outcomes in these patients and their families. This report also highlights the role of consanguinity in genetic disorders and the importance of genetic counseling. Further research to gain deeper insight into TUSC3 gene mutations and appropriate treatment strategies is needed.
